# Feasibility and safety of ALPPS procedure: our experience

**DOI:** 10.1007/s00464-025-12193-3

**Published:** 2025-09-29

**Authors:** S. Caringi, A. Delvecchio, M. Dezio, A. Casella, V. Ferraro, R. Filippo, M. Stasi, S. Marini, R. Calbi, R. Inchingolo, T. M. Manzia, M. Tedeschi, R. Memeo

**Affiliations:** 1 Unit of Hepato-Biliary and Pancreatic Surgery, “F. Miulli” General Hospital, Acquaviva delle Fonti, 70021 Bari, Italy; 2 Department of Surgery, Università Degli Studi Roma “Tor Vergata”, Via Montpellier 1, 00133 Rome, Italy; 3 Department of Radiology, “F. Miulli” General Hospital, Acquaviva delle Fonti, 70021 Bari, Italy; 4 Unit of Interventional Radiology, “F. Miulli” General Hospital, Acquaviva delle Fonti, 70021 Bari, Italy; 5 Department of Surgery Sciences, Transplant and HPB Unit, University of Rome Tor Vergata, Rome, Italy; 6 Department of Medicine and Surgery, LUM University, Casamassima, 70010 Bari, Italy

**Keywords:** ALPPS, Robotic liver resection, Open liver resection

## Abstract

**Background:**

The associating liver partition and portal vein ligation for staged hepatectomy (ALPPS) is a surgical strategy for patients with advanced liver tumors and inadequate future liver remnants (FLR). This study compares post-operative outcomes between open (oALPPS) and robotic (rALPPS) ALPPS in our institution.

**Methods:**

This retrospective monocentric study includes eleven patients who underwent ALPPS procedure between January 2023 and February 2024. Demographics, tumor characteristics, and intra- and post-operative outcomes were collected and analyzed.

**Results:**

In the first-stage ALPPS, the frequency of intermittent hilum clamp and the estimated blood loss were higher in the oALPPS group, while operative time and complications were similar, and total hospital stay was higher in the rALPPS group. In the second-stage ALPPS, the data regarding intraoperative and post-operative data were similar. In rALPPS, there were no post-discharge complications, readmissions, or deaths within 30 days.

**Conclusion:**

This single-center study has an exploratory setting given the small cohort, but if confirmed in larger studies, rALPPS could be a viable alternative to oALPPS.

**Supplementary Information:**

The online version contains supplementary material available at 10.1007/s00464-025-12193-3.

The associating liver partition and portal vein ligation for staged hepatectomy (ALPPS) procedure is a useful strategy to treat patients with advanced primary or metastatic liver tumors and inadequate future liver remnants (FLR) [1]. Since it was first described in 2012, ALPPS has led to excellent results regarding the increase in FLR but at the same time, high morbidity and mortality rates [2]. Patient selection and restriction of indications have subsequently led to better outcomes [3, 4] such that ALPPS has become an accepted and established procedure worldwide [5]. At the same time, minimally invasive liver surgery and robotic surgery, in particular, have exploded. It is now more than proven how this type of approach positively impacts the post-operative outcome while maintaining excellent safety profiles [6, 7]. This monocentric study aims to analyze the post-operative outcomes of ALPPS performed in our center and, subsequently, to compare the outcomes of the open ALPPS (oALPPS) group with the robotic ALPPS (rALPPS) group.

## Methods

**Fig. 1:**
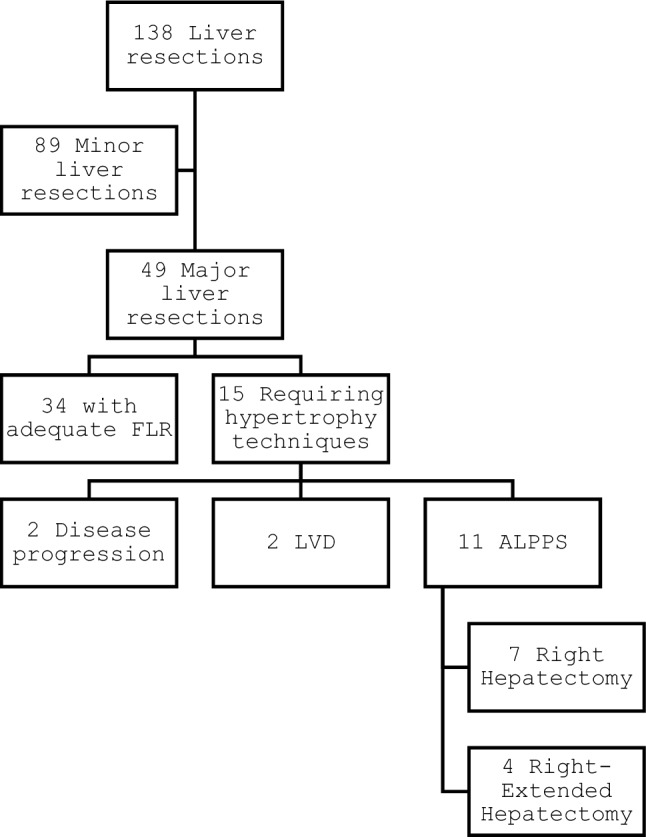
Flowchart of patients included in the study.

This retrospective monocentric study was conducted at the Unit of Hepato-Biliary and Pancreatic Surgery of the "F. Miulli" General Hospital, Italy, between January 2023 and February 2024. During these fourteen months, one hundred and thirty-eight patients underwent liver resection due to primary or secondary liver cancer. Among these, forty-nine were major liver resections, of which fifteen required hypertrophy techniques to achieve an adequate FLR. Among these fifteen, in two cases, there was disease progression between the two stages, and in two cases, we preferred a liver venous deprivation (LVD) due to a very low FLR. In conclusion, there were eleven ALPPS performed in our center during the study period (Fig. 1), of which seven were right hepatectomies and four right-extended hepatectomies.

Initially, the decision whether to perform the surgery with an open or robotic approach was made during the weekly surgical board. Subsequently, as experience increased, ALPPS were all performed robotically. Preoperative and interstage volumetric assessment was done in collaboration with the radiology departments. All patients underwent preoperative volumetric analyses based on the computed tomography (CT) scan. After stage 1, FLR hypertrophy was routinely assessed after 10–15 days. If hypertrophy was inadequate, reassessment was done almost every 10 days. The aim was to reach an FRL ≥30% of volume in case of normal liver, ≥40% for severe steatosis, cholestasis, and previous chemotherapy, and ≥50% in cirrhotic patients [8]. Stage 2 was always decided after a multidisciplinary evaluation of FLR hypertrophy, liver function tests, and the general status of the patient. All robotic procedures were performed using the da Vinci Xi Surgical System. For each patient, we collected preoperative, intraoperative, and post-operative data from the first stage of ALPPS, intraoperative and post-operative data from the second stage of ALPPS, and follow-up data.

Data before first-stage ALPPS include age, sex, body mass index (BMI), previous surgery (both open and laparoscopic), Charlson Comorbidity Index (CCI) score, American Society of Anesthesiologists (ASA) score, presence of cirrhosis of the liver, underlying pathology, size of the main lesion, and proximity to vessels.

For both the first stage and the second stage of ALPPS, we collected data on the number and duration of the hilum clamps, estimated blood loss, operative time, post-operative complications, and hospital stay. After the first stage, we also analyzed data regarding portal embolisation, hepatic vein embolisation, and portal vein ligation. The data concerning the second-stage ALPPS include an analysis of the type of procedure performed.

Follow-up data concern complications after discharge and readmission within 90 days.

The two subgroups were only compared from a descriptive point of view due to the smallness of the samples, using the median value and the first and third interquartile ranges (IQR).

### Surgical technique

In our experience, the majority of ALPPS procedures are rescue intraoperative strategies or as a salvage option following failure of portal vein embolization (PVE) or liver venous deprivation (LVD). However, ALPPS can also represent a first-line indication in cases of extensive bilateral tumor burden. In such scenarios, the principle is to make the first stage as conservative as possible to facilitate a safer and more manageable second stage.

The robotic approach is generally preferred, depending on patient- and tumor-specific characteristics (Video 1). An open approach is favored in cases with multiple previous surgeries, high bilateral tumor burden, or when an extended right hepatectomy is required.

During the first stage, if bilobar metastases are present, we begin with resection of the tumors in the left liver. The decision between PVE, LVD, or portal vein ligation (PVL) with alcoholization is based on the anticipated difficulty of hilar dissection and its potential impact on the second stage, as well as the volume required to achieve an adequate future liver remnant (FLR). When hilar dissection is technically demanding or could complicate the subsequent surgery, we opt for PVE, avoiding direct hilar manipulation.

Even with the robotic approach, we tend to favor PVE, as it allows us to avoid extensive hilar dissection and helps minimize adhesions, thus facilitating a safer and more manageable second stage.

LVD is reserved for patients with an FLR <20–25%, where a significant volume increase is needed.

We use some tricks to facilitate the second stage, such as

- Leave a polypropylene suture thread around the right hepatic artery to speed up its identification;
- Adopt an anterior approach with partial liver transection, which helps minimize the risk of biliary leakage. The depth of transection depends on the required hypertrophy.
- Avoid liver mobilization to reduce post-operative adhesions;
- Use hemostatic devices along the transection line and place a surgical drain into the transection site to limit fluid collections

If PVL is not performed during the first stage, interventional radiology performs PVE or LVD during the same hospital stay. Patients are then discharged and re-evaluated using a CT scan and liver volumetry as previously described.

During the second stage, we begin with cholecystectomy. Liver mobilization is performed either at the beginning or end, depending on the chosen anterior approach. In cases with large tumors or dense adhesions, we favor the anterior approach. The hepatic hilum is dissected to identify and divide the right hepatic artery and right biliary duct. In cases where PVE has been performed, we additionally isolate and divide the right portal vein branch during hilar dissection. Parenchymal transection is then completed, followed by the identification and division of the right hepatic vein and the middle hepatic vein if an extended right hepatectomy is planned. The procedure ends with liver mobilization, reconstruction of the falciform ligament, and placement of a drain.

ALPPS steps are shown in Table 1 and in Video 1.

**Table 1:** ALPPS Steps

**First Stage**	
**PVL** Left liver tumor resection in case of bilateral metastasis Hilum dissection Right Portal vein ligation Right hepatic artery identification and looping Partial parenchymal transection	**PVE/LVD** Left liver tumor resection in case of bilateral metastasis Partial parenchymal transection Post-operative PVE/LVD
**Second stage**	
Cholecystectomy Liver mobilization Right hepatic artery division Right biliary duct division Parenchymal transection completion Right/Middle Hepatic vein division	

*PVL* portal vein ligation, *PVE* portal vein embolization, *LVD* liver venous deprivation

## Results

Patients’ characteristics and perioperative data are shown in Table 2.

**Table 2:** Patients’ characteristics and perioperative data

Variables	Total *n* = 11	oALPPS *n * = 6	rALPPS *n * = 5	*p * value
Age (yr), median (IQR)	68 (60**–**75)	62 (53.75**–**73.25)	73 (67**–**75)	0.15
Female, *n (%)*	6 (54.5)	4 (66.7)	2 (40)	0.43
BMI (kg/ m2), median (IQR)	25.7 (24.6**–**26.7)	26.7 (25.9**–**28)	24.4 (23.7**–**25.3)	0.05
ASA score ≥ III, *n* (%)	4 (36.4)	1 (16.7)	3 (60)	0.18
Charlson comorbidity index score, median (IQR)	7 (6**–**8)	7.5 (7**–**8)	6 (4**–**10)	0.60
Previous open abdominal surgery, *n* (%)	6 (54.5)	3 (50)	3 (60)	0.77
Previous laparoscopic abdominal surgery, *n* (%)	4 (36.4)	2 (33.3)	2 (40)	0.84
Cirrhosis, *n* (%)	2 (18.2)	0	2 (40)	0.18
Tumor: size of the biggest lesion (mm), median (IQR)	45 (31**–**65)	53 (34-67.5)	36 (32**–**45)	0.56
Vessel contact, *n* (%)	5 (45.5)	2 (33.3)	3 (60)	0.43

*BMI* body mass index, *ASA* American Society of Anesthesiologists, *MELD* mayo end-stage liver disease.

The median age was higher in the rALPPS group, but these data are not statistically significant (p value 0.15), and 54.5% were female patients (66.7% in the oALPPS group vs. 40% in the rALPPS group). The median BMI was 25.7 kg/m^2^ (IQR: 24.6–26.7 kg/m^2^ ) but in the oALPPS group was 26.7 kg/m^2^ (IQR: 25.9–28 kg/m^2^ ) and in the rALPPS group 24.4 kg/m^2^ (IQR: 23.7–25.3 kg/m^2^), a statistically significant difference (p value: 0.05). The 36.4% of patients had an ASA ≥ III, mostly in the rALPPS group (60% vs. 16.7%), and the median Charlson comorbidity score mean was 7 (IQR: 6–8) with a non-statistically significant difference between the two groups (*p* value: 0.6). A total of seven patients (63.6%) had previous abdominal surgery. In particular, six patients (54.5%) had previous open abdominal surgery (50% oALPPS vs. 60% rALPPS) and four patients (36.4%) had minimally invasive abdominal surgery (33.3% oALPPS vs. 40% rALPPS). Three patients (27.3%) had both open and minimally invasive abdominal surgery. As shown in Table 1, two patients (18.2%) had liver cirrhosis, one HCV-related and one from metabolic syndrome, and both of them belong to the rALPPS group. The median size of the biggest lesion was 45 mm (IQR: 31–65 mm), higher in the oALPPS group (53 mm, IQR: 34–67.5 mm) rather than in the rALPPS group (36 mm, IQR: 32–45 mm), and in 45.5% of the cases, there was a lesion with vessel contact (33.3% oALPPS vs. 60% rALPPS). However, none of these data appear to have statistical significance.

The indication for surgery was due to colorectal liver metastasis (CRLM) in five cases (45.4%), while in the other six cases (54.6%), with an equal distribution, the indications were for hepatocellular carcinoma (HCC), neuroendocrine tumor metastasis (NETLM), and cholangiocarcinoma (CCA).

Data regarding the first-stage ALPPS are reported in Table 3.

**Table 3:** First-stage ALPPS intraoperative and post-operative data

Variables	Total *n* = 11	oALPPS *n* = 6	rALPPS *n* = 5	*p* value
Hilum clamp, median (IQR)	3 (1**–**6)	6 (3**–**7)	1.5 (0.75**–**2.5)	0.09
Hilum clamp time (minutes), median (IQR)	46 (18–70)	71 (38–86)	25 (8–54)	0.16
Estimated blood loss (ml), median (IQR)	350 (113–475)	500 (300–500)	50 (50–400)	0.07
Operative time (minutes), median (IQR)	280 (265–320)	300(275–336.25)	280 (230–295)	0.19
Portal vein ligation, *n (%)*	3 (27.3)	2 (33.3)	1 (20)	0.66
Portal vein embolization, *n (%)*	8 (72.7)	4 (66.7)	4 (80)	0.66
Hepatic vein embolization, *n (%)*	3 (27.3)	2 (33.3)	1 (20)	0.66
Post-operative complications, *n (%)*	2 (18.2)	1 (16.7)	1 (20)	0.9
Severe complications (Clavien-Dindo ≥3), *n (%)*	0	0	0	
Total hospital stay (days), median (IQR)	8 (6–9)	7 (5.25–8.75)	9 (7–9)	0.28

The clamping of the hepatic hilum occurred a median of 3 times (IQR: 1–6 times), much less in the rALPPS group (1.5, IQR: 0.75–2.5 times vs. 6, IQR: 3–7 times), and with a median total duration of 46 minutes (IQR: 18–70 minutes), higher in the oALPPS group (71 minutes, IQR: 38–86 minutes vs. 25 minutes, IQR: 8–54 minutes) but not statistically significant. The median estimated blood loss was 350 ml (IQR: 113–475 ml), higher in the oALPPS group (500 ml, IQR: 300–500 ml vs. 50 ml, IQR: 50–400 ml), while operative time was 280 minutes (IQR: 265–320 minutes), similar between the two groups. None of these data, however, have statistical significance.

Going into details, in addition to partial parenchymal transection, in the oALPPS group, in 4 cases, multiple wedges were performed in the left lobe, and in 1 case, the one performed for cholangiocarcinoma, locoregional lymphadenectomy was performed. In the rALPPS group, on the other hand, multiple wedges of the left lobe were performed in 2 cases.

Most of the time (72.7% of the total cases, 66.7% in the oALPPS group, and 80% in the rALPPS group), a portal vein embolization was performed, while a portal vein ligation was performed in the remaining cases. Hepatic vein embolization was performed in 27.3% of cases (33.3% in the oALPPS group vs. 20% in the rALPPS group). There were post-operative complications in 18.2% of cases (16.7% in the oALPPS group vs. 20% in the rALPPS group, *p* value 0.9) but never severe (Clavien–Dindo ≥3). Specifically, in the oALPPS group, one patient with COPD from cigarette smoking required oxygen therapy for the first two days post-surgery. In the rALPPS group, on the other hand, a patient with an Apfel Score of 2 required antiemetics in the first 24 hours post-surgery. The mean hospital stay was 8.4 days (6.8 days in the oALPPS group vs. 10.4 days in the rALPPS group)

Data regarding second-stage ALPPS are reported in Table 4.

**Table 4:** Second-stage ALPPS intraoperative and post-operative data

Variables	Total *n* = 11	oALPPS *n* = 6	rALPPS *n* = 5	*p* value
Surgical procedure performed • Right hepatectomy, *n (%)* • Extended right hepatectomy, *n (%)*	7 (63.6) 4 (36.4)	3 (50) 3 (50)	4 (80) 1 (20)	
Hilum clamp, median (IQR)	1 (1–2)	1 (1–2)	1 (1–1.25)	0.57
Hilum clamp time (minutes), median (IQR)	18 (12–24)	15 (14–20)	20 (11–25)	0.67
Estimated blood loss (ml), median (IQR)	500 (250–675)	500 (400–600)	500 (100–700)	0.9
Operative time (minutes), median (IQR)	280 (345-473)	350 (345–471.25)	390 (380–435)	0.89
Post-operative complications, *n (%)*	4 (36.3)	2 (33.3)	2 (40)	0.84
Severe complications (Clavien-Dindo ≥3), *n (%)*	0	0	0	
Total hospital stay, median (IQR)	8 (7–12)	8 (7–11.25)	8 (6–12)	0.83

Of the eleven procedures performed, seven (63.6%) were right hepatectomies and four (36.4%) extended right hepatectomies. In the oALPPS group, they were carried out equally, while in the rALPPS group, a right hepatectomy was performed in 80% of the cases and an extended right hepatectomy in 20% of cases.

The clamping of the hepatic hilum occurred a median of 1 time (IQR: 1–2 times), similar between the two groups, with a median total duration of 18 minutes (IQR: 12–24 minutes), 15 minutes (IQR: 14–20 minutes) in the oALPPS group, and 20 minutes (IQR: 11–25 minutes) in the rALPPS group. Estimated blood loss and operative time were similar in the two groups, as well as post-operative complications and hospital stay. None of the intraoperative and post-operative second-stage data were therefore statistically significant.

About post-operative complications, in addition to the same complications as in the first stage (oxygen therapy for the COPD patient in the oALPPS group and antiemetics for the patient in the rALPPS group), there was one patient with minor bleeding in the oALPPS group, treated conservatively and with the transfusion of one unit of red blood cells (ISGLS grade A), and one patient with ascites in the rALPPS group, treated with albumin and diuretics (ISGLS grade B).

At a mean follow-up of 17 months, there were 2 cases of disease recurrence in the oALPPS group and 2 cases in the rALPPS group. In particular, in the oALPPS group, one patient with metastases from lung NETs had a disease recurrence in the bone nine months after the second stage, treated with radiotherapy. Another patient in the oALPPS group with liver metastases from colorectal carcinoma had a recurrence of disease at the liver level one year after the second stage, treated with an atypical resection. In the rALPPS group, on the other hand, a patient operated on for cholangiocarcinoma had a peritoneal recurrence six months after the second stage and, despite chemotherapy, died three months after the diagnosis of the recurrence. Another patient in the rALPPS group with hepatocellular carcinoma had a hepatic recurrence 17 months after the second stage, treated with TACE and immunotherapy.

### Literature review

The origins of ALPPS and its initial dissemination trace back to Germany. In 2007, Prof. H.J. Schlitt inadvertently performed the first in situ split [9]. The case involved a 49-year-old male patient with perihilar cholangiocarcinoma scheduled for a right trisectionectomy. During surgery, the left bile duct was divided at the base of the round ligament for frozen section analysis. The right portal vein was also divided, and a full-thickness parenchymal transection was performed along the falciform ligament. At this point, concerns arose regarding the adequacy of the FLR, prompting the surgical team to halt the procedure while preserving the left hepatic artery, venous drainage, and biliary flow. The operation was concluded with a hepaticojejunostomy. A follow-up CT scan one week later demonstrated significant hypertrophy of the FLR, allowing the completion of the right trisectionectomy on post-operative day 8.

This innovative approach was initially termed "in situ split liver resection."

The original technique for right trisectionectomy involved two stages: the first stage included right PVL and in situ splitting of the liver parenchyma along the right side of the falciform ligament while retaining the arterial, biliary, and venous structures of the right liver. When bilateral tumors were present, left lobe tumor resection was performed during this stage. After sufficient FLR hypertrophy, typically achieved within nine days, the second stage consisted of right trisectionectomy, with ligation of the right hepatic artery, right bile duct, and hepatic veins.

This technique rapidly gained traction in Germany, and in 2011, Dr. H. Lang from Mainz formally described it at the EAHPBA Congress in Cape Town [10], presenting a series of three cases.

In 2012, Schnitzbauer et al. [1] published the first large series of 25 cases across five German centers. The technique quickly gained recognition as a groundbreaking approach with the potential to transform liver surgery.

Later in 2012, de Santibañes and Clavien [11] introduced the acronym "ALPPS" (Associating Liver Partition and Portal Vein Ligation for Staged Hepatectomy). That same year, following the IHPBA Congress in Paris, Clavien, Lang, and Santibañes established the International ALPPS Registry. In 2015, during the first International Expert Meeting in Hamburg, eight key recommendations were formulated regarding the technique, indications, and standardization of terminology.

At the 12th EAHPBA Congress in Mainz in 2017, the 10th anniversary of ALPPS was celebrated, focusing on the current status and future perspectives of the procedure.

The initial rapid spread of ALPPS was tempered by reports of high morbidity and mortality rates in early series.

Stage 1 of the procedure was recognized as particularly complex and aggressive, prompting the adoption of technical modifications aimed at reducing complications and making the surgery less invasive.

**Fig. 2:**
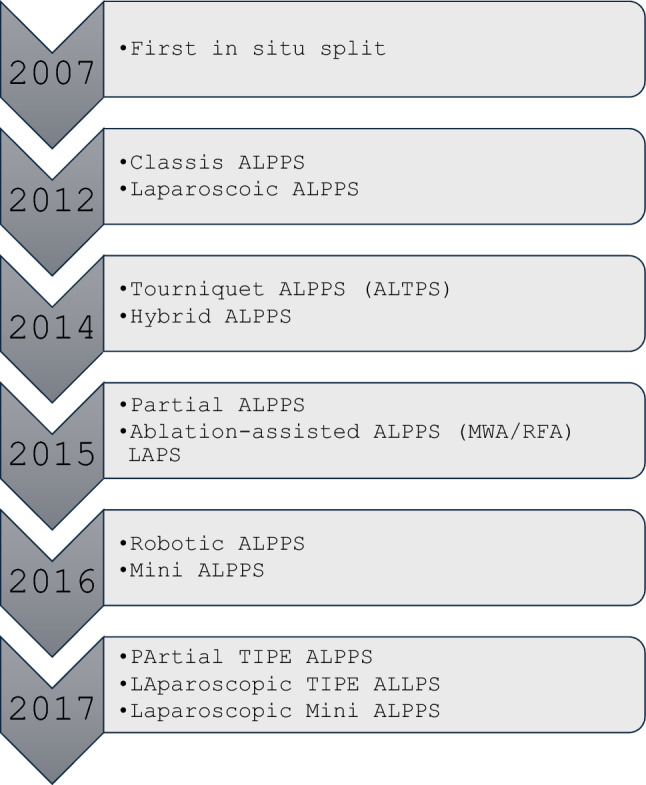
Timeline APPS with most important variants.

Over time, several modifications of the original technique have been developed to optimize outcomes and facilitate the second stage:

- Minimally Invasive ALPPS (2012): Initially applied to Stage 1 and later extended to the entire procedure [12, 13].
- Tourniquet ALPPS, ALTPS (2014): Robles et al. [14] proposed this technique, which replaces in situ splitting with a tourniquet applied along the transection line, reducing operative time during Stage 1.
- Hybrid ALPPS (2014): Li et al. [15] proposed a three-step, non-touch approach with in situ liver splitting, right PVE one day later, and second-stage hepatectomy.
- Radiofrequency/Microwave ALPPS, LAPS (2015): PVL creates a "necrotic groove" as a virtual partition [16].
- Partial ALPPS (2015): Described by Petrowsky et al. [17], involving partial (50-80%) rather than complete parenchymal transection (Fig. 2). This technique is associated with zero mortality, reduced morbidity, and comparable FLR hypertrophy.
- Mini ALPPS (2016): A combination of partial parenchymal transection and intraoperative PVE [12].
- Robotic ALPPS (2016): The first fully robotic ALPPS was performed in Spain [18].
- TIPE ALPPS (2017): Introduced transileocolic portal vein embolization [19].

In 2016, Linecker et al. [20], following the first ALPPS Expert Meeting in Hamburg, proposed a standardized terminology to enable consistent comparisons of different ALPPS variations (Table 5). This standardized terminology facilitates communication among surgeons, optimizes comparisons between techniques, and promotes further advancements in the field of liver surgery.

**Table 5:** ALPPS Terminology

Rescue ALPPS	ALPPS was performed because of inadequate hypertrophy after PVE should be labeled
PVE-ALPPS	The intentional use of PVE as part of the first stage
Stage 1-Laparoscopic/Robotic ALPPS	Laparoscopic/Robotic stage 1, but open surgery for stage 2
*Stages 1 and 2-Laparoscopic/Robotic ALPPS*	Laparoscopic/Robotic for both stages 1 and 2
Partial ALPPS	Incomplete transection
Tourniquet ALPPS Radiofrequency ALPPS Microwave ALPPS	Various techniques of parenchymal division

This classification was based on four principles:

- Simple and self-explanatory terms
- Alignment with the Brisbane classification of liver anatomy
- Avoidance of new acronyms and neologisms
- Use of prepositions to indicate variations, structured as follows: strategy, stage, access, PVE if used, transection, and type of hepatectomy

## Discussion

The associating liver partition and portal vein ligation for staged hepatectomy (ALPPS) procedure has been accepted and established worldwide [4], and some studies show that it is an effective and safe procedure even when performed with a robotic approach [21, 22]. In this case series, we analyzed our experience with ALPPS, performed initially with the open technique and later with the robotic technique. All cases were previously evaluated in our multidisciplinary meeting, where the indication for surgery was given. Remarkably, in 81.9% of cases, the surgical indication was given for metastatic disease or for cholangiocarcinoma, diseases that, to date, are only treated with liver transplantation within clinical trials [23, 24]. The cases performed for HCC (18.1%), on the other hand, were not candidates for liver transplantation due to comorbidities and age [25]. On average, indeed, patients were elderly according to the World Health Organization definition [26] and had a one-year mortality risk of 85% in accordance with the Charlson Comorbidity Index score [27]. Concerning intraoperative differences, the analysis of our data regarding the first-stage ALPPS shows that in the robotic group, less clamping of the hepatic hilum is performed, resulting in a shorter hepatic hypoperfusion time. This finding, together with the lower blood loss estimated in the rALPPS group, is probably due to the technologies available to the robotic system, both in terms of image display and coagulation energy. Reducing these factors is crucial in liver surgery as it has been shown that intraoperative hepatic hypoperfusion by intermittent clamping of the hepatic hilum can influence the post-operative outcome [28]. However, our data concerning the post-operative period of the first-stage ALPPS in the robotic group appear to be worse than in the corresponding open group in terms of complications and total hospital stay. These findings may be influenced by the higher mean age and ASA score observed in patients undergoing rALPPS compared to those in the oALPPS group. Furthermore, in the case of robotic surgery, we typically perform portal vein embolization after surgery by interventional radiologists, thereby physiologically increasing the length of hospital stay. However, there have been no severe post-operative complications (Clavien-Dindo ≥3) in either the first-stage ALPPS or the second-stage procedures, regardless of the surgical approach used. The data concerning second-stage ALPPS are almost comparable between the two groups in terms of time to liver hypoperfusion, estimated blood loss, operative time, post-operative complications, and total hospital stay. However, these findings contrast with those of the first stage, reflecting the significantly greater technical and surgical complexity of the second stage. Second-stage ALPPS is inherently more demanding due to the presence of adhesions, altered anatomy, and the need for precise vascular and biliary dissection. For these reasons, very few centers perform the second stage using a robotic approach, and the current literature remains limited, with scarce data and studies available on this topic.

### Limitations

Limitations of this study include the retrospective design and the small sample under examination.

## Conclusions

Robotic ALPPS is a very demanding procedure, especially in the second stage. Robotic platforms have made it possible to perform very demanding surgeries in a minimally invasive manner, including ALPPS itself, but to date, it remains a procedure performed in only a few centers worldwide. Our single-center study aims to analyze the preoperative, intraoperative, and post-operative data of a patient population undergoing ALPPS at a time when we had the possibility of performing this surgery with both techniques. The results, although from a small cohort, show the non-inferiority of the robotic technique compared to the open technique for this surgery, leaving the feeling that robotic ALPPS can be a procedure with excellent safety profiles if performed by experienced robotic hepatobiliary surgeons in high-volume centers.

## Supplementary Information

Below is the link to the electronic supplementary material.

Supplementary file1 (MP4 299930 KB)

## Data Availability

The data presented in this study are available upon request from the corresponding author due to privacy reasons.
